# Highly Sensitive Electrochemical Biosensor Using Folic Acid-Modified Reduced Graphene Oxide for the Detection of Cancer Biomarker

**DOI:** 10.3390/nano11051272

**Published:** 2021-05-12

**Authors:** Renu Geetha Bai, Kasturi Muthoosamy, Rando Tuvikene, Huang Nay Ming, Sivakumar Manickam

**Affiliations:** 1 Nanotechnology Research Group, Department of Chemical and Environmental Engineering, Faculty of Engineering, University of Nottingham Malaysia, 43500 Semenyih, Malaysia; rgeetha@tlu.ee (R.G.B.); Kasturi.Muthoosamy@nottingham.edu.my (K.M.); 2 School of Natural Sciences and Health, Tallinn University, 10120 Tallinn, Estonia; rantuv@tlu.ee; 3 School of Energy and Chemical Engineering, New Energy Science & Engineering, Xiamen University Malaysia, 43900 Sepang, Malaysia; huangnayming@xmu.edu.my; 4 Petroleum and Chemical Engineering, Faculty of Engineering, Universiti Teknologi Brunei, Bandar Seri Begawan BE1410, Brunei

**Keywords:** folic acid, folate receptor, folate targeted, electrochemical sensor, cancer biomarker, rGO-FA, differential pulse voltammetry, biosensor

## Abstract

The detection of cancer biomarkers in the early stages could prevent cancer-related deaths significantly. Nanomaterials combined with biomolecules are extensively used in drug delivery, imaging, and sensing applications by targeting the overexpressed cancer proteins such as folate receptors (FRs) to control the disease by providing earlier treatments. In this investigation, biocompatible reduced graphene oxide (rGO) nanosheets combined with folic acid (FA)-a vitamin with high bioaffinity to FRs-is utilized to develop an electrochemical sensor for cancer detection. To mimic the cancer cell environment, FR-β protein is used to evaluate the response of the rGO-FA sensor. The formation of the rGO-FA nanocomposite was confirmed through various characterization techniques. A glassy carbon (GC) electrode was then modified with the obtained rGO-FA and analyzed via differential pulse voltammetry (DPV) for its specific detection towards FRs. Using the DPV technique, the rGO-FA-modified electrode exhibited a limit of detection (LOD) of 1.69 pM, determined in a linear concentration range from 6 to 100 pM. This excellent electrochemical performance towards FRs detection could provide a significant contribution towards future cancer diagnosis. Moreover, the rGO-FA sensing platform also showed excellent specificity and reliability when tested against similar interfering biomolecules. This rGO-FA sensor offers a great promise to the future medical industry through its highly sensitive detection towards FRs in a fast, reliable, and economical way.

## 1. Introduction

Folic acid (FA) or pteroylmonoglutamic acid is an important B-group vitamin involved in various metabolic pathways regulating foetal development, genetic material synthesis, and ageing [1,2]. Being an essential nutrient, it is involved in the vital ‘one-carbon transfer reaction’ occurring in most of the body’s metabolic pathways [3,4,5]. However, mammalian cells are incapable of producing FA, and of avoiding the nutritional deficiency, exogenous intake of this nutrient to cells is necessary. According to the Food and Drug Administration (FDA), daily FA intake should be in the range of 100–1000 µg [6]. Compared to normal cells, the proliferating cells exhibit enhanced FA consumption due to their significant contribution to biosynthetic pathways. This strongly supports the link of FA to cancer [7]. Due to its structure and pH sensitivity, the intake of this nutrient to cells is highly limited. In normal cells, the uptake of FA occurs via two major mechanisms, which involve cellular endocytosis. The transportation of FA occurs either via reduced folate carrier proteins or via the membrane-bound folate receptor (FR) proteins. While the FR proteins fulfil the usual requirement of FA in normal cells, overexpression of FR in cells is often associated with malignancy and cancer [3,8,9,10,11,12].

FA possesses different types of interactions with the body proteins. FRs and folate-binding proteins (FBP) have a high affinity towards FA. FBP are ~30 kDa glycoprotein with 222 amino acids. These FBP regulate the trafficking and homeostasis of folate inside the body. FR is bound to the outer surface of the cells via a glycosylphosphatidylinositol (GPI) anchor. In human, FR exists in multiple isoforms such as FR-α, FR-β, FR-δ, and FR-γ. Only FR-α and FR-β possess GPI anchors, which aid easy recognition by FA in a sensor [13,14].

FR expression in normal human tissue is tightly regulated. In the amino acid level, FR-α (38 kDa) and FR-β (34 kDa) exhibit 71% identity; however, very less information is available about FR-δ and FR-γ. In normal tissues, FR-α ex­pression is mostly limited to the apical plasma membrane present in kidneys and certain epithelial tissues. This limits the accessibility of FR-targeting agents towards the normal cells. In the case of tumour cells, however, epithelial cell depolarisation promotes FR targeting. In epithelial lineage cancers, FR-α expression is remarkably amplified. Overexpression of FR-α in non-mucinous ovarian carcinomas, endometrial, lung, colorectal, paediatric ependymomas, mesotheliomas, and renal cell carcinomas makes it a useful biomarker for tumour identification [15]. Nevertheless, FR-β is associated with myeloid leukaemia and activated macrophages (inflammation and tumours). Hence, FR-β is useful as a biomarker for myeloid leukaemia, tumour-related macro­phages, and inflammatory diseases, like rheu­matoid arthritis or osteoarthri­tis [16,17,18]. This selective expression of FR-α and FR-β ensures their potential as specific biomarkers for the targeted delivery of imaging and therapeutic agents to different types of cancer and other abnormalities in the human body. Antibodies of FR-α and FR-β also act as promising therapeutic candidates for tumour-targeted therapy [19,20,21,22,23]. 

FRs are cysteine-rich glycoproteins that mediate FA intake through a receptor-mediated endocytosis process. When FA binds to FR, the part of the cell membrane containing FR-FA forms a vesicle, folding inwards in the cell, forming an endosome. Afterwards, the endosome dissociates from the cell wall by acidification and FA releases from FR to the internal cell compartments. Inside the cells, FA is processed at low pH. During the FA processing, FR will be returned to the cell surface in a recycling manner [24]. The X-ray crystallographic structure of FR-α was recently detected, revealing the complex molecular structure of the FA–FR interaction [25].

Although FRs are membrane-bound proteins, overexpressed FRs in body fluids are proved in many earlier investigations. For example, increased FRα levels are found in the blood of ovarian cancer patients [26]. Similarly, in multiple myeloma, overexpression of FRα and FRβ is observed in the blood [11]. Additionally, in most cancers, when cancer progresses to metastasis stage, local infiltration and migration of the tumour cells to nearby tissues and subsequently to the circulatory system is often observed, supporting this test scenario using the body fluids [27]. 

Due to FR’s overexpression in various cancers, FA-targeted cancer therapy is widely explored in the current nanomedical research fields [28,29]. The successful targeted delivery of genetic material, drugs, imaging agents, nanoparticles etc., ensures the potential of FR targeting in future medical scenario [30,31,32,33,34]. Among the variety of tumour targeting agents, FA is of low molecular weight, inexpensive, and possesses high stability and non-immunogenicity compared with other targeting proteins, peptides, or antibodies [35]. Having a great potential for diagnosis and therapy, these FR-targeted systems could be advantageous to nanotheranostic applications. Currently, FA-based theranostic systems are widely explored in both in vitro and in vivo investigations focusing on better treatments for cancer [32,36,37,38,39]. 

The limitless potential of FA-based targeting for cancer cells has attracted sensing applications, too [40,41,42,43,44]. Considering the high bioaffinity, FA could be utilized as a bio-recognition element in developing an electrochemical sensor for FR. The unique interaction of FA and FR will result in electron transfer blockage by the insulating cell membrane. In electrochemical sensing, cyclic voltammetry and impedance spectroscopy were used to study the interaction between the FR present on the cells and the FA functionalized transducer [45]. Being a biomolecule, FA possesses negligible electrical responses. To enhance the sensor electrical properties, a transducer material is often functionalized with FA. Due to the high affinity of FA (K_D_~10^−10^ M), the conjugation with a carrier system does not alter its affinity towards FR binding, enabling efficient targeting of FA-based systems in the drug delivery applications [46]. Similarly, the introduction of a transducer material will not influence the targeting properties of FA. The various transducer nanomaterials involved in sensing application were gold nanoparticles, carbon nanoparticles, gold-polymer combinations, etc. Due to the excellent electronic and electrochemical properties, graphene-based systems become an attractive transducer element in electrochemical sensors [31,47,48].

Graphene or reduced graphene oxide (rGO) is a two-dimensional carbon material with unique chemical and physical properties. Graphene-based materials are widely employed in drug/gene delivery, biosensing, and photothermal therapy applications [49,50]. In addition to excellent conductivity, graphene-based materials have advantages such as easy functionalization, biocompatible nature, and good stability in physiological conditions, making it a versatile transducer material [51,52,53,54,55,56,57]. Moreover, due to the high surface area and rich π conjugation structure, graphene-based structures offer an excellent platform for the effective loading of bio-recognition elements [58]. Based on our previous investigation, rGO prepared by a green approach was highly biocompatible, readily dispersible in water, and exhibited high electrical conductivity [59,60]. FA conjugation to graphene-based material follows the chemical linkages using N-hydroxysuccinimide (NHS) alone [61], 1-ethyl-3-(3-dimethylaminopropyl)carbodiimide-N-hydroxysuccinimide (EDC/NHS) by covalent reactions [62,63] or by the aid of polymeric linkers [64]. 

Compared to other sensors, electrochemical sensing is fast, easy, specific, reliable, and economical. In addition, an electrochemical sensor operates in a non-destructive manner with multi-detection capabilities. These real-time analytical sensors are fundamental sensors that can be further developed to produce an easy-to-handle, comparatively portable, reproducible, sensitive, and accurate sensing device. The current electrochemical cell consists of a three-electrode system. A glassy carbon (GC) electrode is used as a reusable working electrode modified with the sensing nanomaterial [65,66]. 

## 2. Scope of the Study

Despite the effective localization of tumours in vivo or in vitro for targeted drug/gene/imaging agent delivery, few studies have reported the electrochemical detection of cancer cells using FA. However, in our study for the sensor development, we utilized a biocompatible rGO nanocomposite green synthesized by *Ganoderma lucidum* (G.l.) extract. Herein, utilizing FA targeting property combined with the excellent electrocatalytic potential of the rGO nanosheets is explored as the electrochemical sensing platform for the real-time analysis of FR for cancer diagnosis application. This rGO-FA electrochemical sensor could be utilized to detect increased FRs in the body fluids as early detection for cancer progression. Thus, it can be used to monitor and control the progression of diseases from their advanced forms. It is a fast, reliable, and economical means of detecting cancer easily. FR is utilized in this model study to mimic the cancer cell environment. The concentration of FR can be expressed in molar units since in cancer cells, FR is usually found in picomolar. For instance, a study by Doucette et al. [67] explains that the FR levels in the case of JAR cells were found to be in the range of 4–26 pmol/mg protein, and in the case of Caco-2 cells, the FR levels were found to be in the range of 0.2–1.5 pmol/mg protein. Similarly, for MA−104 cells, the FR levels were found to be in the range of 0.3–17 pmol/mg protein. Considering these FR ranges, the limit of detection in the current study is 1.69 pM, significant and comparable with previous findings. 

## 3. Experimental Section

### 3.1. Materials

*Ganoderma lucidum* (G.l.) was procured from Ganofarm Sdn. Bhd. (Tanjung Sepat, Malaysia). Graphite powder was received from Asbury Graphite Mill Inc. (Asbury, NJ, USA), FR- β, dimethylsulfoxide (DMSO). Ferricyanide, phosphate buffer solution (PBS), bovine serum albumin (BSA), human serum albumins (HSA), and sodium hydroxide were purchased from Sigma-Aldrich, USA. Milli-Q water from the Millipore water purification system (EMD Millipore, Billerica, MA, USA) was used in all the experiments. All the chemicals used were of analytical grade unless otherwise stated.

### 3.2. Synthesis of rGO

Initially, GO was synthesized from graphite flakes by modified Hummer’s method [68]. rGO was prepared using G.l. extract following our previously reported protocol [59]. Briefly, an equivalent amount of GO (0.1 mg/mL) and G.l. extract was stirred at 60 °C for up to 12 h, followed by multiple washing with distilled water. 

### 3.3. Synthesis of rGO-FA

FA was dissolved in DMSO and transferred to an aqueous solution of rGO at pH 7 (1:10 ratio). FA and rGO were allowed to react by stirring for 12 h at 37 °C. Black boxes were used during the reaction to prevent any possible light exposure. After the reaction, the solution was centrifuged at 10,000 rpm for 5 min to remove free FA, which was not bound to rGO. The produced rGO-FA was then freeze-dried.

### 3.4. Characterization Techniques

The UV-Vis absorption spectra of rGO, FA, and rGO-FA nanocomposite were investigated using aqueous solutions of the samples in a Lambda 35 Spectrophotometer (Perkin Elmer, Waltham, MA, USA). The morphological studies using dried samples were carried out using field emission scanning electron microscopy Quanta SEM 400 instrument (Quanta 400 FEI, OR, USA), high-resolution transmission electron microscopy (HRTEM, Philip model JOEL, Tokyo, Japan), and atomic force microscopy Agilent Technologies AFM System (Agilent Technologies, Santa Clara, CA, USA) using ultra-sharp tip (non-contact high resonance frequency, nanosensor probe). The crystalline properties of the dried samples were examined using X’Pert Pro diffractometer (XRD, PANalytical, Almelo, the Netherlands), with CuKα radiation and a step size of 0.001° (2θ). The presence of specific chemical bonds and functional groups were examined from the characteristic vibrations and corresponding peaks in the range of 4000–400 cm^−1^ using a Fourier transform infrared (FTIR) spectrometer (Spectrum RX1, Perkin Elmer, TX, USA) using the dried samples mixed with KBr spectroscopic grade Potassium bromide (KBr) as the window material to make pellets to analyze the IR-based vibrations. Elemental composition was determined using X-ray photoelectron spectroscopy (XPS, Kratos Axis Ultra, Shimadzu, Japan) using Al Kα monochromatic radiation at 1486.6 eV with a step size of 0.1 eV and 20 eV pass energy.

### 3.5. Electrochemical Measurements

The electrochemical studies were performed using a PAR-VersaSTAT-3 electrochemical workstation (Princeton Research, TN, USA). The studies were conducted on a three-electrode electrochemical cell system at room temperature (RT). The glassy carbon (GC) electrode was used as a working electrode; platinum and silver/silver chloride (Ag/AgCl) electrodes were used as counter and reference electrodes, respectively. Before modification, the GC electrode was cleaned by polishing with 0.03-micron alumina slurry and distilled water, followed by continuous potential cycling between +1 and −1 V in 0.1 M H_2_SO_4_. The modified electrode was fabricated by drop-casting 5 μL of aqueous rGO-FA nanocomposite solution on GC electrode surface and allowed to dry at room temperature for 1 h. A total of 0.1 M [Fe(CN)_6_]^3−/4−^ solution prepared in PBS solution (pH 7.4) was used as the supporting electrolyte for the electrochemical experiments, and all the potentials were estimated against Ag/AgCl reference electrode unless otherwise indicated. 

## 4. Results and Discussion

**Figure 1:**
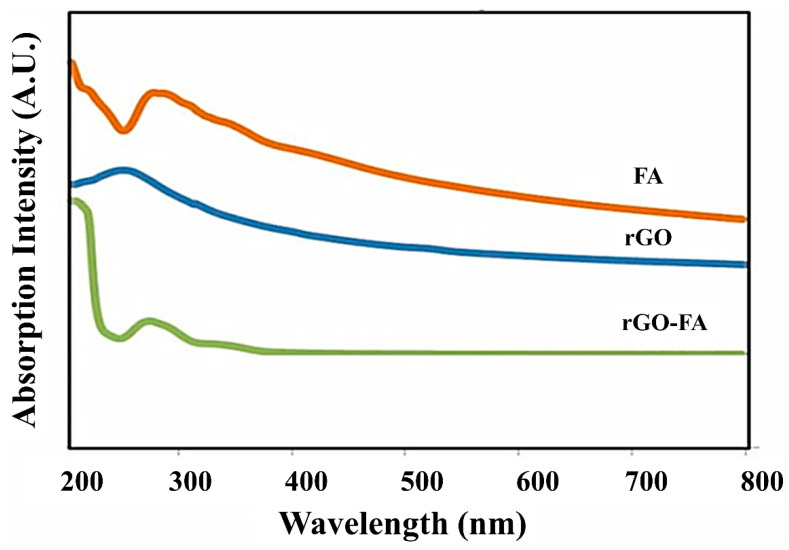
UV-Vis spectroscopic analysis of rGO (0.1 mg/mL), FA (0.1 mg/mL), and FA-conjugated rGO (rGO-FA) (0.1 mg/mL) in aqueous solutions. The red shift of the rGO-FA spectra was observed compared to the spectra of rGO and FA alone.

UV-Vis spectroscopic analysis of rGO, FA, and rGO-FA resulted in characteristic absorption peaks, as shown in Figure 1. The peak at 260 nm in rGO is a red-shifted peak from the characteristic peak of GO at 230–240 nm. In FA, the UV-Vis spectrum varied depending on the system’s pH [69]. In our investigation, FA at pH 7 exhibited a sharp peak at 280 nm. rGO-FA exhibited a slight blue shift with a sharp peak positioned at 276 nm corresponding to FA deposited on rGO nanosheets. Similarly, the small hump appearing around 300–350 nm could be the red-shifted peak of rGO. Peak shift in nanomaterials is associated with changes in the size of the material. Here, similar to polymerization, the conjugation of FA molecules on the surface of rGO resulted in the change of size and the slight shift in the absorption peaks [70,71]. The FA is directly attached to the rGO sheets by π–π interaction.

**Figure 2:**
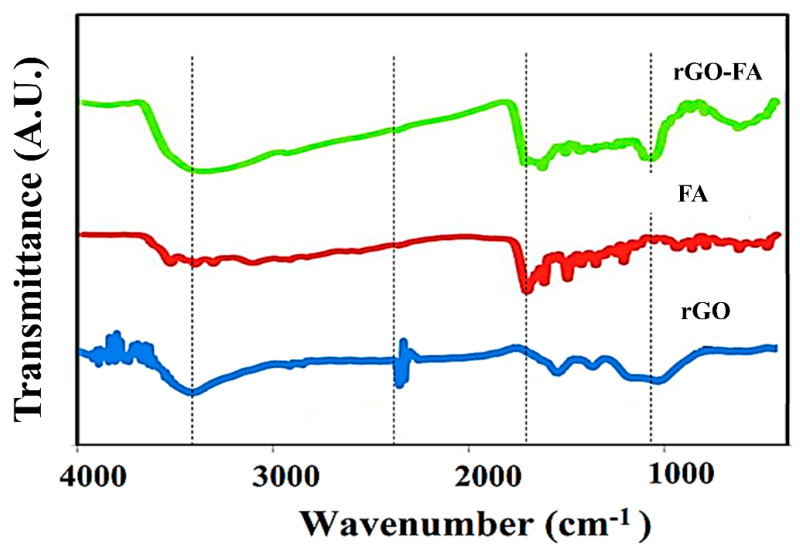
FTIR spectroscopic analysis of dried forms of rGO, FA, and rGO-FA using spectroscopic grade KBr-based pellets.

In Figure 2, the water-mediated synthesis approach of rGO, FA, and rGO-FA exhibited peaks corresponding to the -OH stretching vibrations at 3400 cm^−1^. rGO and rGO-FA showed peaks at 1400 cm^−1^ which could be due to the C=C stretching of aromatic groups. Similarly, FA and rGO-FA exhibited peaks at 1640 cm^−1^ corresponding to the C=O of carboxylic acid. The presence of peaks in rGO and rGO-FA at 1100 cm^−1^ is due to the C-O stretching vibration of carboxylic groups [59]. The multiple characteristic peaks of FA from 1600 to 1500 cm^−1^ correspond to different functional groups, such as C=O, C=N, C=C, and NH bending [72], and other observed peaks, as well as the corresponding functional groups, are listed in Table S1. In rGO-FA, the presence of similar peaks with slight red shifting confirms the conjugation of FA on rGO’s surface. 

**Figure 3:**
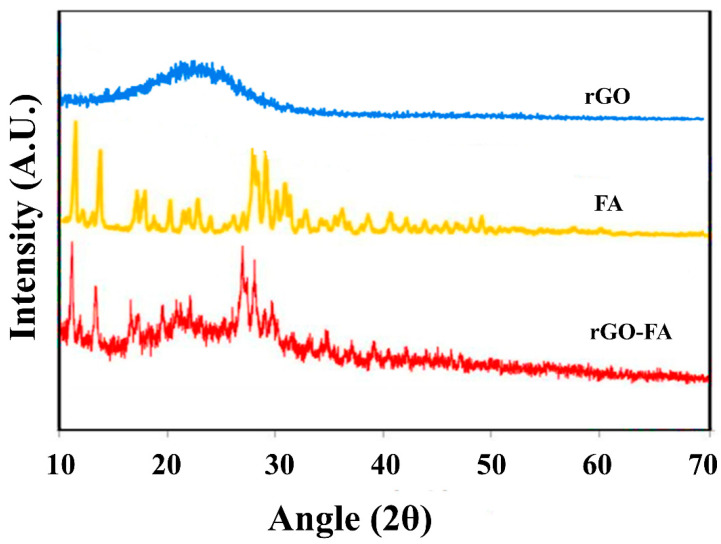
XRD analysis of dried rGO, FA, and rGO-FA.

The XRD analysis of rGO showed a characteristic peak shown in Figure 3 at an angle of 22° with (002) crystal plane. XRD analysis of FA exhibited multiple diffraction peaks at 2θ values of 11°, 13°, 19°, 23°, 27°, 29°, 31°, 35°, 40°, and 45°, in agreement with the FA standard JCPDS files 42-1963 and 29-1716 [73,74]. In rGO-FA, similar FA peaks were observed with a slight shift in the 2θ position. Additionally, rGO-FA showed a broad peak at 21° corresponding to the (002) crystal plane of rGO.

**Figure 4:**
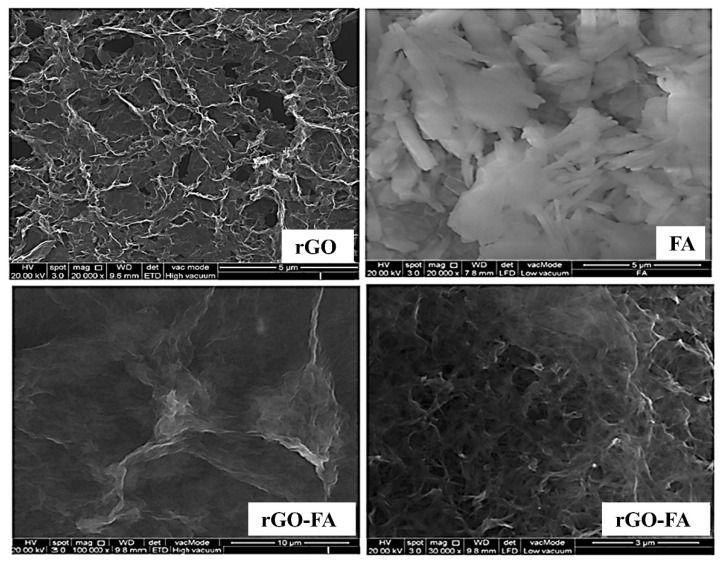
SEM images of rGO, FA, and rGO-FA at lower (left) and higher (right) magnifications.

SEM images provided a 2D morphology analysis of the rGO, FA, and rGO-FA nanocomposite (Figure 4). rGO appeared crumbled with the stacking of few layers, showing the effective conversion of GO to rGO during the reduction. The images of FA exhibit flake-like morphology with signs of agglomeration. The SEM images of rGO-FA show homogenous dispersion of FA on the surface of rGO. In the magnified image of rGO-FA, the transparent layers of rGO could be observed, which shows signs of interruption to the prior stacking due to the effective incorporation of FA in between the rGO layers. This confirms the successful loading of FA on rGO layers.

**Figure 5:**
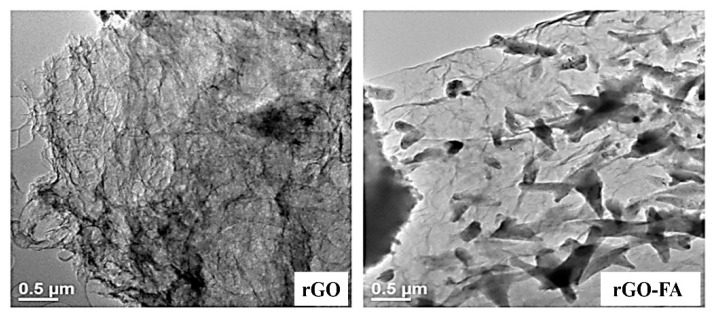
HRTEM images of rGO and rGO-FA.

A detailed morphological analysis was carried out by HRTEM (Figure 5), where the rGO appeared wrinkled with signs of stacking of few layers, agreeing to the SEM images of rGO. In rGO-FA, transparent rGO sheets could be observed with FA uniformly distributed on the rGO sheets. FA appears as flower-like structures on the surface of the rGO sheets that facilitate and maintain the few-layer structure of rGO by preventing the rest of the exfoliated layers.

To analyze the 3D profile/thickness of the rGO-FA nanocomposite, AFM analyses were carried out. rGO-FA showed a height profile of <5 nm and based on our earlier report, the thickness of rGO produced was <2 nm [59], which supports the effective loading of FA on the surface of rGO (Figure S1). This finding is also in agreement with the other morphological characterization. 

EDAX analysis confirms the elemental composition of rGO, FA, and rGO-FA. As shown in Table 1, the weight % of C and O are 59.41 and 40.59, respectively, for rGO. In FA, the weight % of C, N, and O are 62.26, 28.21, and 9.53, respectively. A similar weight % pattern was observed for rGO-FA with C, N, and O of 68.70, 18.05, and 13.25, respectively. 

The XPS spectra of the rGO-FA could be used to identify the elemental composition of rGO-FA after FA binding (Figure S2). The wide scan analysis revealed the atomic % values of C1s, O1s, and N1s as 67.36, 18.65, and 13.99, respectively. The existence of C-C, C-O, C=O, and COO bonds reveals either rGO or FA. Moreover, other bonds were also observed corresponding to N interaction with O and C, specifically NC=O bonds positioned at 401 eV and N1s peak at 399 eV corresponding to C=N. These N bonds could be contributed to by the conjugation of FA and rGO [75].

The electrochemical properties of rGO-FA were evaluated by cyclic voltammetry (CV) using the three-electrode system with glassy carbon (GC) as working electrode, Ag/AgCl as reference and platinum as the counter electrode. A redox couple [Fe(CN)_6_]^3−/4−^ was used as the electrolyte for various analysis. This electrolyte was prepared in 0.1 M PBS solution (pH 7.4) containing 0.1 M KCl and 2 mM [Fe(CN)_6_]^3−/4−^. 

**Figure 6:**
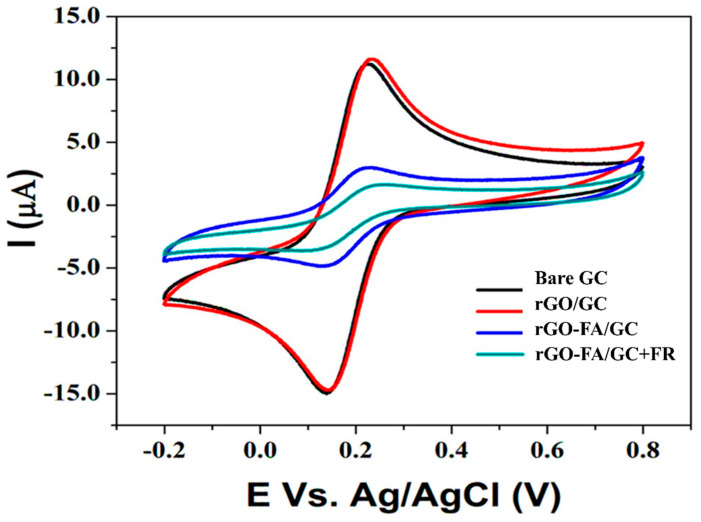
CV analysis of bare GC, rGO/GC, rGO-FA/GC, and rGO-FA/GC + 10 nM FR in 0.1 M [Fe(CN)_6_]^3−/4−^ at a scan rate of 50 mV s^−1^.

A stepwise modification was observed in the CV analysis of the modified electrodes compared to the bare GC, as shown in Figure 6. Changes in the peak-to-peak separation and associated anodic and cathodic current responses represented the charge transfer barriers in the electron transfer kinetics of [Fe(CN)_6_]^3−/4−^. The decrease in the redox couple’s peak currents could be due to the insulating behaviour of the biomolecules [76]. Compared to the bare GC and rGO, FA’s non-covalent functionalization on the rGO showed a decrease in the current in [Fe(CN)_6_]^3−/4−^. FR’s introduction to the system promotes effective interaction of FA with FR, and the current flow was significantly reduced. A decrease in the current corresponds to the effective interaction of FA and FR. Upon adding FR, an insulating layer was formed on the surface of rGO-FA/GC, causing a blockage to the interfacial electron transfer and thus resistance to electron flow [77]. 

**Figure 7:**
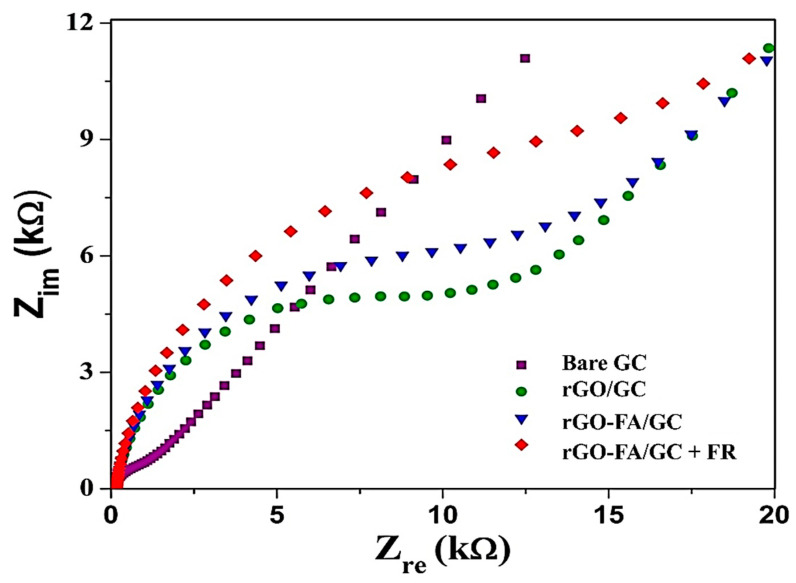
EIS spectra of bare GC, rGO/GC, rGO-FA/GC, and rGO-FA/GC + 10 nM FR in 0.1 M [Fe(CN)_6_]^3−/4−^ at a frequency range of 10 Hz–100 kHz.

The electrochemical impedance spectroscopy (EIS) analysis is a sensitive method in detecting the interfacial changes in the impedance of electrodes with response to the addition of biomolecules or living cells such as macrophages, endothelial cells, fibroblasts, bacterial cells, or cancer cells. Upon cells’ addition, an insulating effect is developed on the surface of these cells [78]. The EIS curve has two portions: a semicircle and a linear section. The semi-circular curve at a higher frequency corresponds to the electron transfer process, and the semicircle diameter is calculated as the electron transfer resistance, R_et_. The linear portion at low frequencies denotes the diffusion process [76]. EIS spectra of different GC modified electrodes in Figure 7 show the resistance developed on the electrode-electrolyte interface during the charge transfer. The bare GC exhibits the least resistance to charge transfer, and rGO-FA shows the highest resistance in charge transfer. This demonstrates the effective π–π interaction of rGO and FA, which prevents electron flow. Finally, upon the addition of FR to the rGO-FA-modified electrode, the bio-recognition of FR takes place at the modified electrode, which further prevented the charge transfer at the electrode-electrolyte interface.

**Figure 8:**
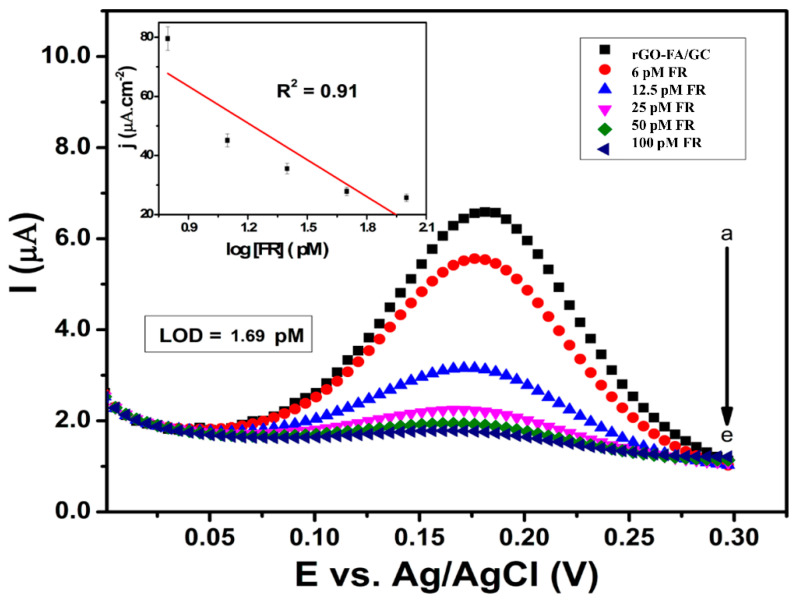
DPV analysis of rGO-FA with the addition of FR at the predetermined intervals at a scan rate of 50 mV s^−1^.

Due to the complexities of redox reactions at the biomolecular level, CV analysis for detecting FR using the modified rGO-FA/GC gave negligible current responses. Though EIS was widely used for live cell-based sensor analysis, previous studies reported an extreme change in the resistance by extending the analysis duration [76]. This could be due to the cells’ possible detachment, caused by prolonged electric field associated cell death. Thus, in the current study, the detection mode of analysis was switched to differential pulse voltammetry (DPV) instead of a CV to obtain a faster and reliable response. Interestingly, DPV analysis was very sensitive towards the detection of FR. As shown in Figure 8, the highly sensitive detection of FR was conducted via DPV by introducing FR-β at regular intervals of 60 s. The addition of FR showed a significant reduction in the peak current response due to the FA–FR interaction. 

The DPV analysis in the concentration range of 6−100 pM (Figure 8) resulted in a linear relationship between peak current density (j) and log FR concentration with a correlation coefficient, R^2^ = 0.9101. The calibration plot of peak current vs concentration of FR from 6 to 100 pM resulted in a linear equation with I (mA) = 4.638−0.037 [FR] (pM) with a detection limit of 1.69 pM (at an S/N ratio of 3), with milliseconds response time and sensitivity of 0.037 μA pM^−1^ cm^2^, which is highly comparable to other earlier reported detection systems (Table 2).

Conventionally, protein/antibody/DNA conjugated gold electrodes were utilized for sensitive detection approaches to attain similar LoD values, which are expensive and need special handling [88,89,90]. However, in the current report, the developed rGO-FA-modified GC is a novel system, which is economically feasible, sensitive, and allows rapid detection. Reproducibility of the rGO-FA-modified GC was ensured by three repeated experiments.

**Figure 9:**
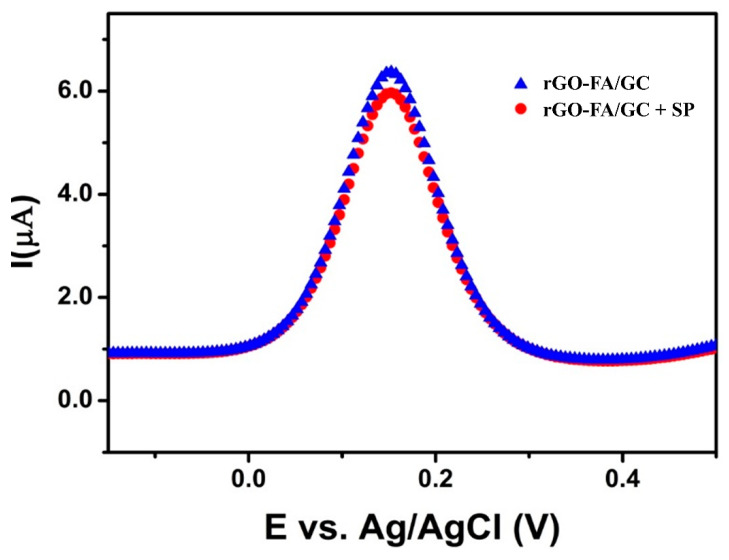
Interference analysis of rGO-FA with the addition of SP/HSA (100 pM) at a scan rate of 50 mV s^−1^.

Interaction of similar interfering biomolecules is a critical concern in sensor development. Under normal conditions, human serum is free of FR. However, during cancer progression or macrophage associated inflammations, the overexpression of FR, especially FR-β, occurs in the serum. Blood serum involves three major serum proteins (SP): albumins, globulins, and fibrinogen. Among these, human serum albumin (HSA) is the most abundant protein in plasma, existing as <50% of the total SP [91]. Thus, the introduction of HSA could be used as a prominent interference analysis. Upon introducing SP/HSA (100 pM) to the rGO-FA/GC sensor, SP’s interference did not exhibit any significant difference in the current response (Figure 9) compared with the detection signals of FR. This finding facilitates using rGO-FA for the selective and reliable detection of FR even in the presence of interfering biomolecules, and the three repeated experiments ensured reproducibility.

## 5. Conclusions

Recognizing FA’s vital role as a potential biomarker for detecting FR, which is associated with cancer progression and immune response associated inflammations, a nanocomposite was developed by the conjugation of FA with rGO for the sensitive detection of FR. rGO-FA was well characterized using SEM, TEM, AFM, XRD, XPS, EDAX, and FTIR techniques. The electrocatalytic properties of rGO-FA were analyzed utilizing a three-electrode electrochemical cell with an rGO-FA-modified GC electrode via a DPV technique. Upon adding FR, rGO-FA-modified GC showed significant current responses in a linear concentration range from 6 to 100 pM, with a detection limit of 1.69 pM. SP was introduced to the system to analyze the effect of interfering molecules, resulting in no significant current responses. The specificity and reproducibility of the electrode were analyzed repeatedly. Apart from usual sensing studies, replacing gold or platinum working electrode with a GC as the working electrode proves this system’s economic feasibility. Compared to the typical expensive FR detection systems that involve gene/protein/antibodies for the detection of biomolecules, the current rGO-FA electrode offers an economical, fast, and sensitive sensing to detect FR biomarkers. The current novel rGO-FA nanocomposite for FR sensing enunciates a great promise for future cancer detection systems.

## Figures and Tables

**Table 1:** EDAX analysis results of rGO-FA.

Material	Carbon (wt%)	Oxygen (wt%)	Nitrogen (wt%)
rGO	59.41	40.59	-
FA	62.26	28.21	9.53
rGO-FA	68.70	18.05	13.25

**Table 2:** FA–FR-based sensing studies for cancer cell detection.

Sensing Material	Working Electrode	Material or Cell Line Used and Range of Detection	Limit of Detection (FR Conc. or Number of Cells/mL)	Method	Reference
FA-DNA–SWNT	Au	FR (0.01–10 nM)	3 pM	DPV	[79]
FA-DNA	Au	FR (1.0–20.0 ng/mL)	0.3 ng/mL	CV SSA	[80]
CNTs@PDA-FA	GC	HL-60 cells (5 × 10^3^–5 × 10^5^ cells/mL)	5 × 10^2^ cells	EIS	[81]
MPA/(Fc-PEI/SWNT)	Au	HeLa cells (10–10^6^ cells/mL)	10 cells	DPV	[62]
PNT–FA	G	HeLa cells (250–5 × 10^3^ cells/ mL)	250 cells	CV	[82]
PNT–FA	G	FR (8–13 nM)	8 nM	CV	[82]
Au/MUA-FA	Au	HeLa cells (6–10^5^ cells/mL)	6 cells	EIS	[83]
Au-FA	BDD	HeLa cells (10–10^5^ cells/ mL)	10 cells	EIS	[84]
FA-AuNPs	Au	Hela cells (1.3 × 10^5^)	Not indicated	CV	[85]
FA-GSH-GNPs	-	HeLa cells (10–10^5^ cells/mL)	100 cells	Absorbance	[86]
FA/PEI/CMC-G	GC	HL-60 cells (500–5 × 10^6^ cells/ mL)	500 cells	EIS	[76]
FA- MHDA-HT-Fc	Au beads	HeLa cells (10–10^6^ cells/mL)	10 cells	DPV	[87]
rGO-FA	GC	FR (6–100 pM)	1.69 pM	DPV	This work

Au: gold, DNA: deoxyribonucleic acid, SWNT: single-walled carbon nanotubes, SSA: steady-state amperometry, CNTs@PDA: polydopamine-modified carbon nanotubes, MPA: 3- mercaptopropionic acid, G: graphene, Fc-PEI: poly(ethylene imine) functionalized with ferrocene, PNT: peptide nanotube, MUA: 11 mercapto undecanoic acid, NPs: nanoparticles, BDD: boron-doped diamond, PTCA: 3,4,9,10-perylene tetracarboxylic acid, CCG: chemically converted graphene, GNPs: gold nanoparticles, GSH: glutathione, GC: glassy carbon, PEI: polyethyleneimine, CMC: carboxymethyl chitosan, G: graphene, MHDA: mercaptohexadecanoic acid, HT: hexanethiol, Fc: ferrocenyl.

## Supplementary Materials

The following are available online at https://www.mdpi.com/article/10.3390/nano11051272/s1, Figure S1: AFM image of rGO-FA and the height profile at cross-section with average thickness as 5 nm, Figure S2: XPS analysis of rGO-FA, displaying various bonds supporting the conjugation of rGO and FA and elemental analysis as an atomic percentage. Table S1: FTIR peaks of FA and its corresponding functional groups.
