# Supplementary material for: Highly Sensitive Electrochemical Biosensor Using Folic Acid-Modified Reduced Graphene Oxide for the Detection of Cancer Biomarker
Source: Nanomaterials (Basel). 2021 May 12;11(5):1272. doi: 10.3390/nano11051272 (PMC8150695; doi:10.3390/nano11051272)
Supplement: Supplementary file 1 [file nanomaterials-11-01272-s001.zip › nanomaterials-1204632-supplementary.pdf]

nanomaterials

Supplementary Information

Highly Sensitive Electrochemical Biosensor Using Folic Acid-Modified Reduced Graphene Oxide for the Detection of Cancer Biomarker

Renu Geetha Bai [1,2], Kasturi Muthoosamy [1], Rando Tuvikene [2], Huang Nay Ming [3] and Sivakumar Manickam [1,4,*]

[1]Nanotechnology Research Group, Department of Chemical and Environmental Engineering, Faculty of Engineering, University of Nottingham Malaysia, 43500 Semenyih, Selangor, Malaysia; rgeetha@tlu.ee (R.G.B.); Kasturi.Muthoosamy@nottingham.edu.my (K.M.)
[2]School of Natural Sciences and Health, Tallinn University, 10120 Tallinn, Estonia; rantuv@tlu.ee
[3]School of Energy and Chemical Engineering, New Energy Science & Engineering, Xiamen University Malaysia, 43900 Sepang, Malaysia; huangnayming@xmu.edu.my
[4]Petroleum and Chemical Engineering, Faculty of Engineering, Universiti Teknologi Brunei, Bandar Seri Begawan BE1410, Brunei
* Correspondence: manickam.sivakumar@utb.edu.bn

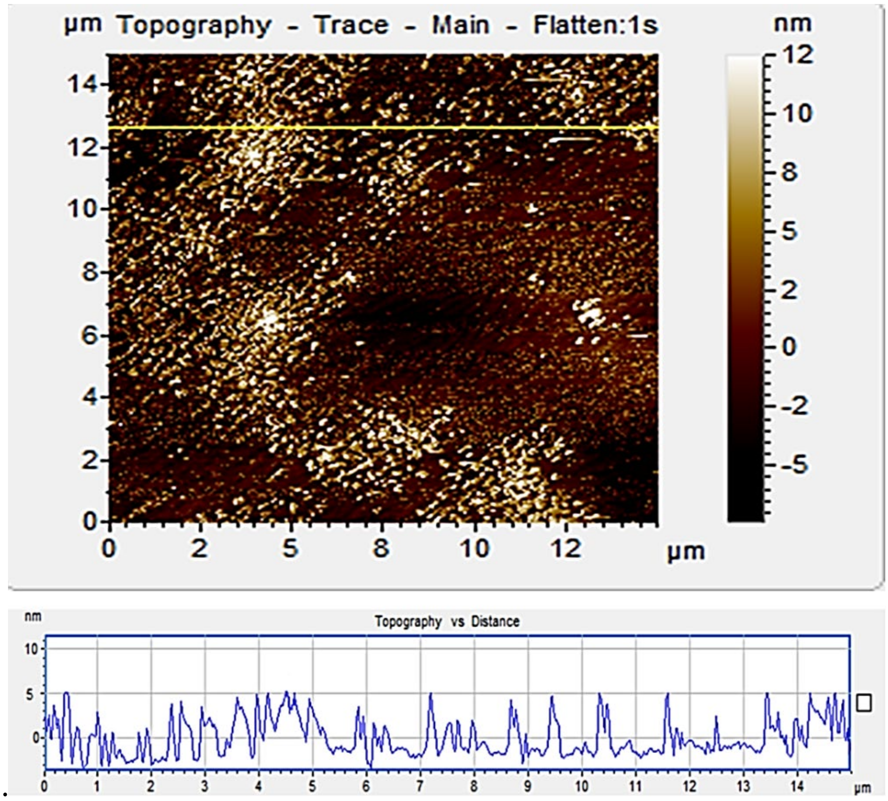

Figure S1. AFM image of RGO-FA and the height profile at cross-section with average thickness as 5 nm.

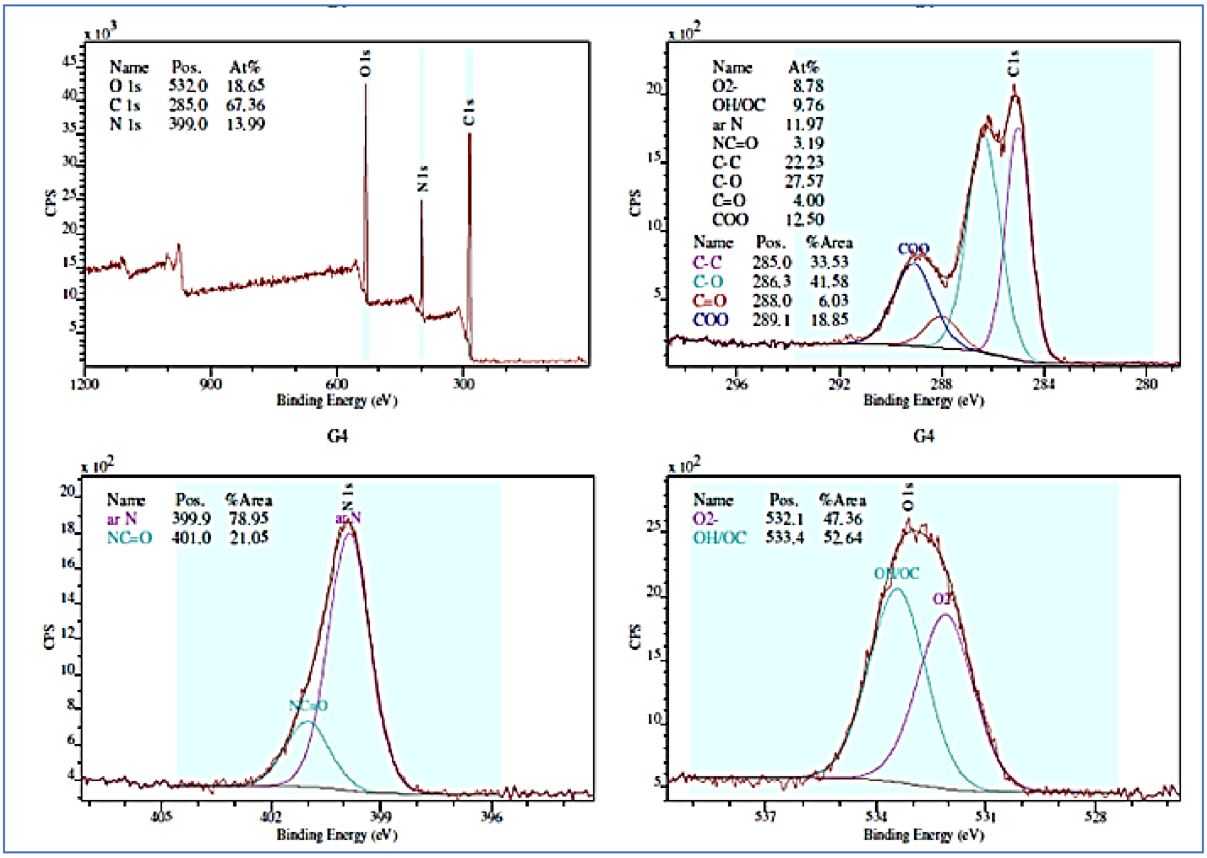

Figure S2. XPS analysis of RGO-FA, displaying various bonds supporting the conjugation of RGO and FA and elemental analysis as an atomic percentage.

Table S1. FTIR peaks of FA and its corresponding functional groups [1]

Wavenumber (cm^{-1})	Functional group
1485–1519	Phenyl and Pterin ring
1604	NH bending
1619	C=C aromatic
1639	C=N
1650	C=O amide
1693	C=O carboxylic
3100–3500	OH carboxylic functional group of the glutamic acid moiety

Reference

1. Mohammed, E.M.K.A.D. Qualitative and quantitative determination of folic acid in tablets by FTIR spectroscopy. *Int. J. Adv. Pharm. Biol. Chem.* **2014**, *3*, 773–780.
